# Supplementary figures and images for: Pyrylium based derivatization imaging mass spectrometer revealed the localization of L-DOPA
Source: PLoS One. 2022 Aug 2;17(8):e0271697. doi: 10.1371/journal.pone.0271697 (PMC9345479; doi:10.1371/journal.pone.0271697)

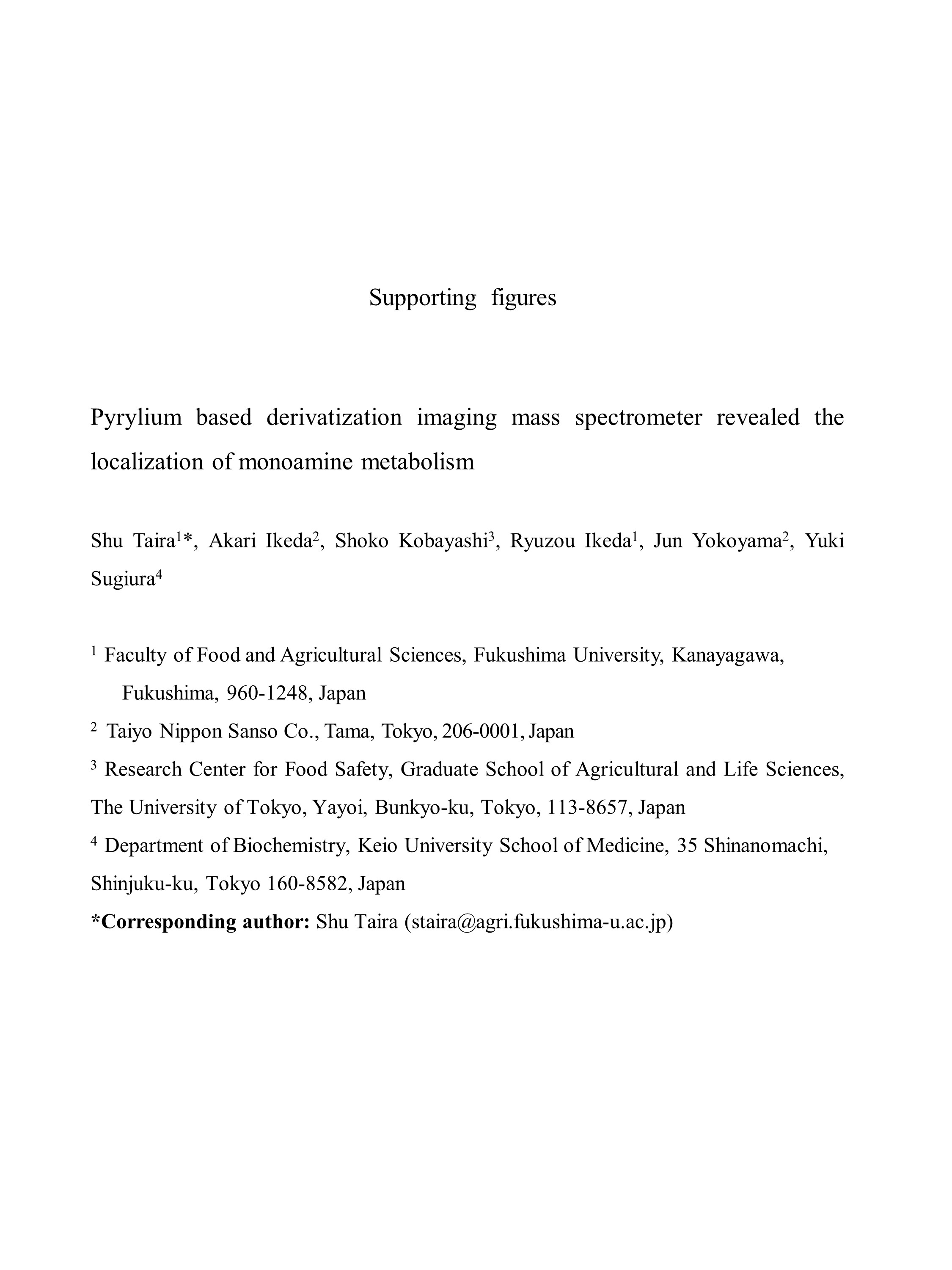

Supplement: S1 File — (ZIP) [file pone.0271697.s001.zip › SI_st.JPG]

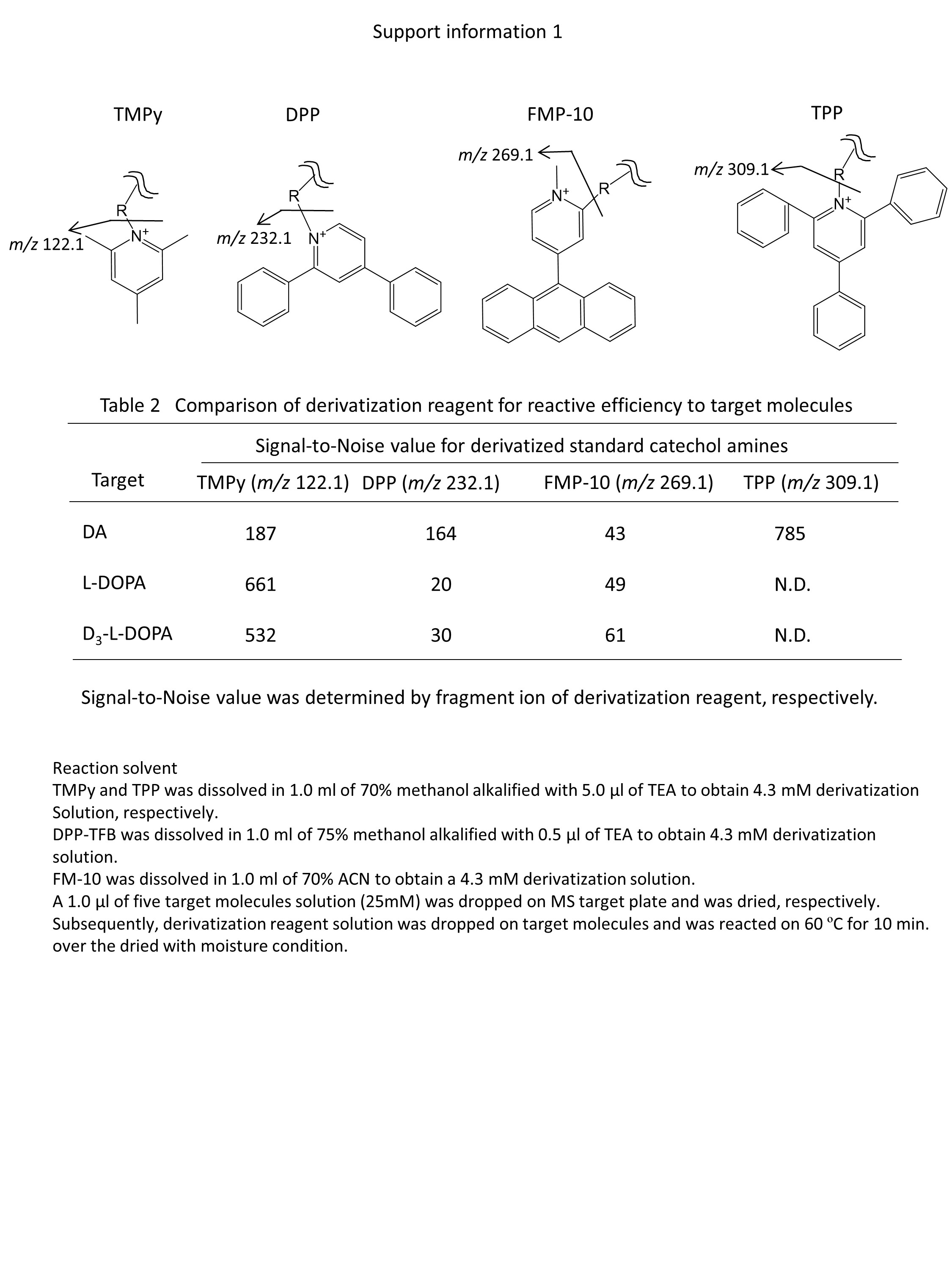

Supplement: S1 File — (ZIP) [file pone.0271697.s001.zip › SI1_st.JPG]

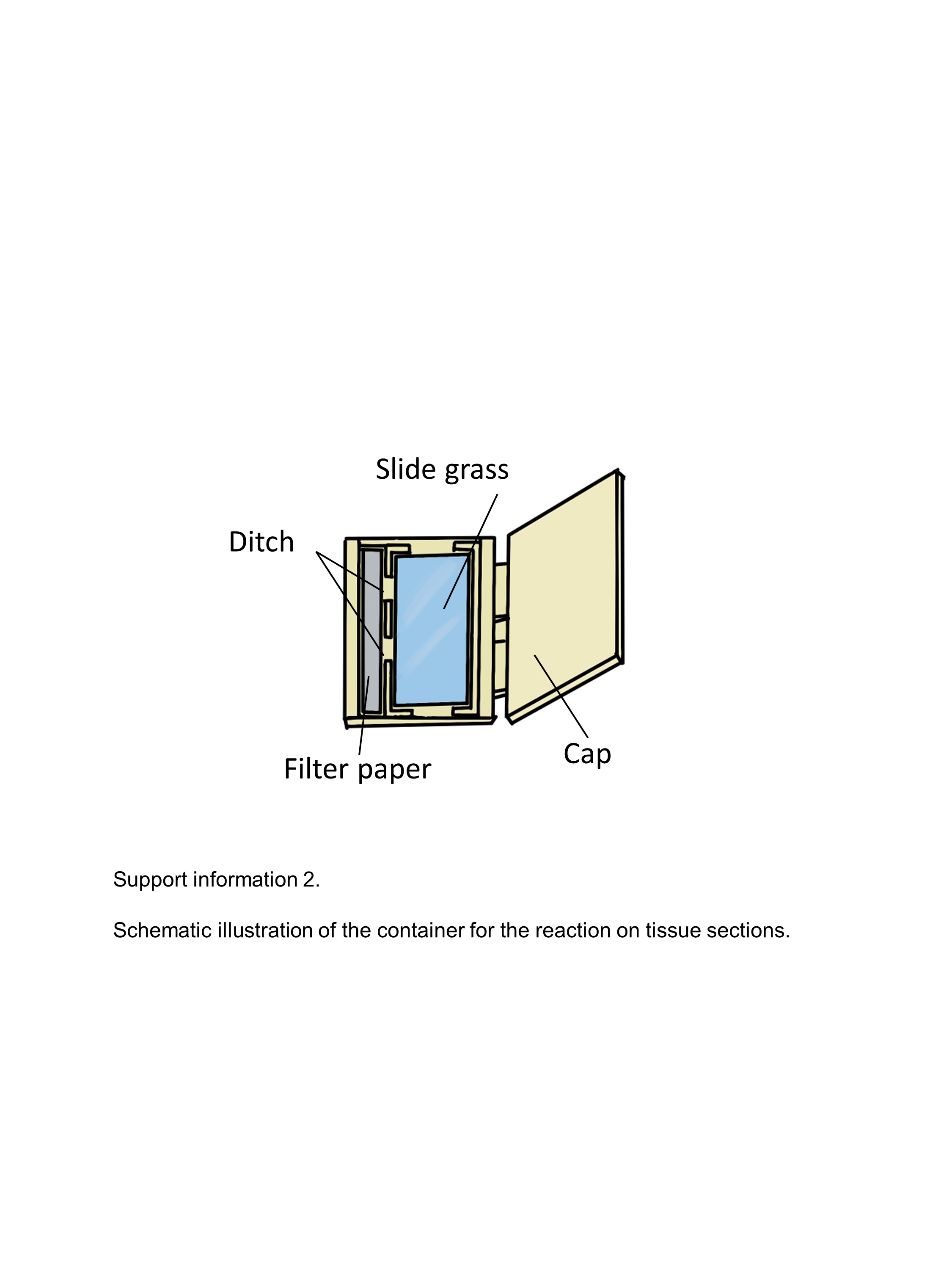

Supplement: S1 File — (ZIP) [file pone.0271697.s001.zip › SI2_st.JPG]

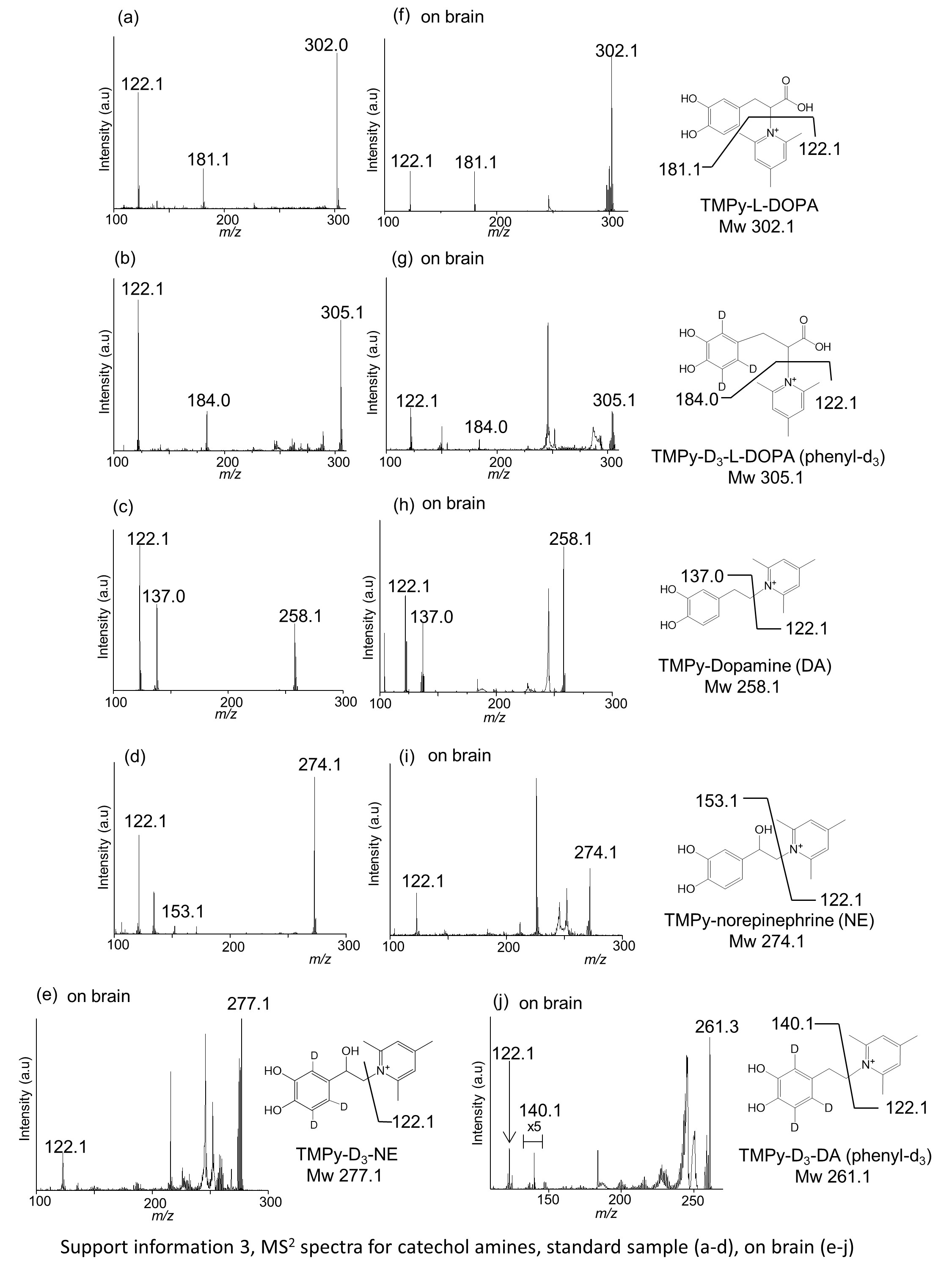

Supplement: S1 File — (ZIP) [file pone.0271697.s001.zip › SI3_st.JPG]

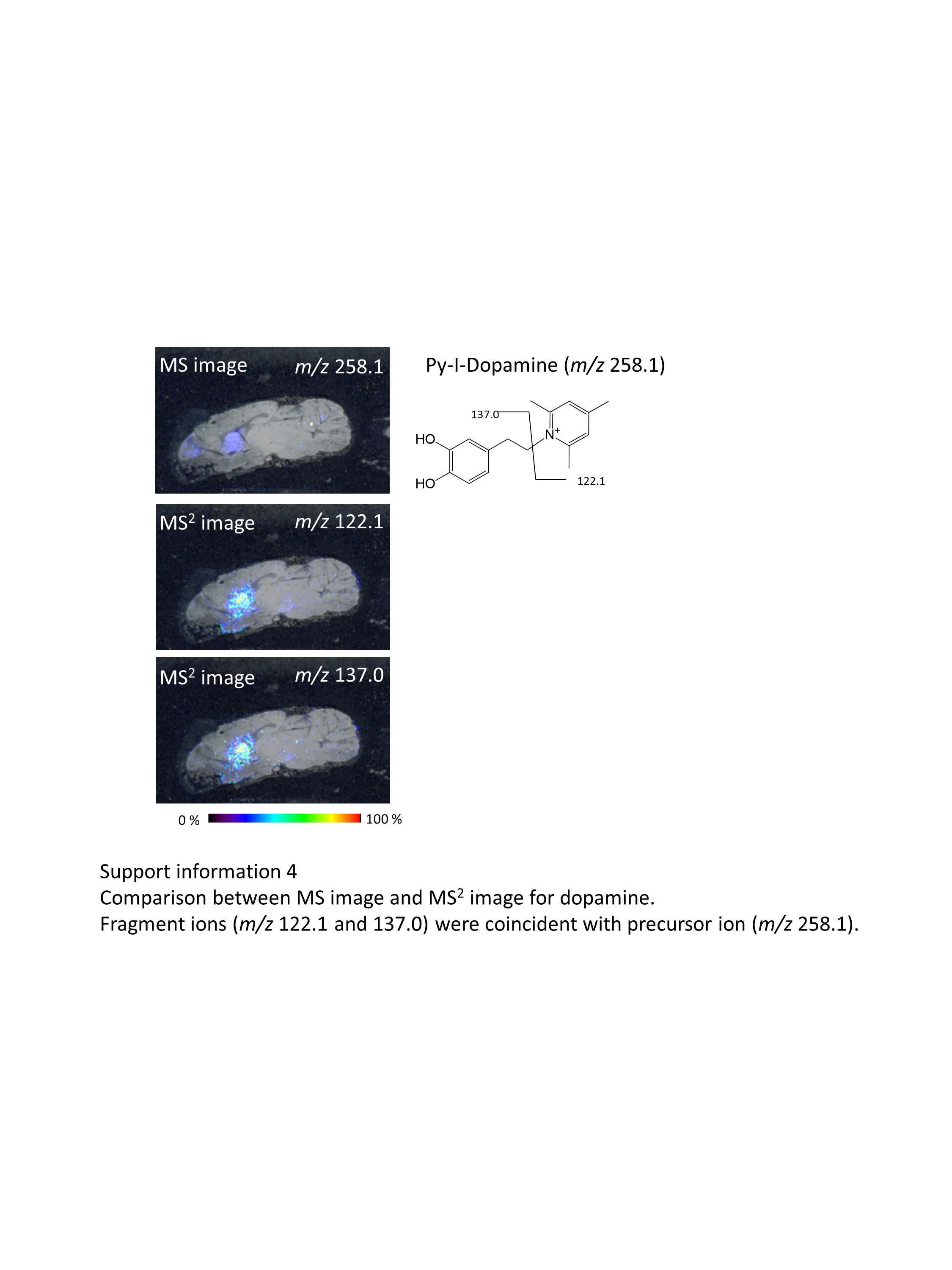

Supplement: S1 File — (ZIP) [file pone.0271697.s001.zip › SI4_st.JPG]
